# Low Adherence to Pneumococcal Vaccination in Lung Cancer Patients in a Tertiary Care University Hospital in Southern Germany

**DOI:** 10.3390/vaccines10020311

**Published:** 2022-02-16

**Authors:** Arno Mohr, Mia Kloos, Christian Schulz, Michael Pfeifer, Bernd Salzberger, Stilla Bauernfeind, Florian Hitzenbichler, Annelie Plentz, Thomas Loew, Myriam Koch

**Affiliations:** 1 Center for Pneumology, Donaustauf Hospital, Ludwigstr. 68, 93093 Donaustauf, Germany; arno.mohr@ukr.de (A.M.); michael.pfeifer@ukr.de (M.P.); 2 Department of Internal Medicine 2, University Hospital Regensburg, Franz-Josef-Strauß-Allee 11, 93053 Regensburg, Germany; mia.kloos@stud.uni-regensburg.de (M.K.); christian.schulz@ukr.de (C.S.); 3 Department of Infection Prevention and Infectious Diseases, University Hospital Regensburg, Franz-Josef-Strauß-Allee 11, 93053 Regensburg, Germany; bernd.salzberger@ukr.de (B.S.); stilla.bauernfeind@ukr.de (S.B.); florian.hitzenbichler@ukr.de (F.H.); 4 Department for Microbiology and Hygiene, University Hospital Regensburg, Franz-Josef-Strauß-Allee 11, 93053 Regensburg, Germany; annelie.plentz@ukr.de; 5 Department for Psychosomatic Medicine, University Hospital Regensburg, Franz-Josef-Strauß-Allee 11, 93053 Regensburg, Germany; thomas.loew@ukr.de

**Keywords:** pneumococcal vaccination, lung cancer, pneumonia, PPSV23, PCV13

## Abstract

Introduction: The aim of this study was to investigate the adherence to vaccinations, especially pneumococcal vaccinations, in lung cancer patients. Methods: the study was performed at the University Hospital Regensburg, Germany. All patients with a regular appointment scheduled between 1 December 2020 and 29 April 2021 and who provided informed consent were included. Available medical records, vaccination certificates, and a questionnaire were analyzed. Results: we included 136 lung cancer patients (NSCLC *n* = 113, 83.1%, SCLC *n* = 23, 16.9%). A correct pneumococcal vaccination according to national recommendations was performed in 9.4% (12/127) of the patients. A correct vaccination was performed for tetanus in 50.4% (66/131), diphtheria in 34.4% (44/128), poliomyelitis in 25.8% (33/128), tick-borne encephalitis in 40.7% (24/59), hepatitis A in 45.5% (7/11), hepatitis B in 38.5% (5/13), shingles in 3.0% (3/101), measles in 50.0% (3/6), pertussis in 47.7% (62/130), influenza in 54.4% (74/136), and meningococcal meningitis in 0% (0/2) of the patients. Conclusion: adherence to pneumococcal vaccinations, as well as to other vaccinations, is low in lung cancer patients.

## 1. Introduction

Lung cancer is one of the most common malignancies worldwide [1,2]. Infectious diseases contribute to morbidity and mortality by delaying appropriate antineoplastic therapy [1]. Vaccinations are an important and effective preventative healthcare measure, especially in patients with chronic diseases [1].

*Streptococcus pneumoniae* (*S. pneumoniae*) is the leading pathogen in community-acquired pneumonia (CAP) and the main cause of lower respiratory infection morbidity globally [3]. Two different vaccines are available for adults: PCV13, a pneumococcal conjugate vaccine containing 13 different capsular types, and PPSV23, a pneumococcal polysaccharide vaccine containing 23 different polysaccharides.

To prevent pneumococcal infections in these patients, vaccinations are almost universally recommended [1,4]. In Germany, the Standing Committee on Vaccination (STIKO) develops national recommendations for the use of vaccines. For patients with lung malignancy, pneumococcal vaccination with PCV13, followed by vaccination with PPSV23 6–12 months later, is recommended; furthermore, a booster dose with PPSV23 should be given after 6 years. The U.S. Centers for Disease Control and Prevention even recommend sequential pneumococcal vaccination and a booster dose with PPSV23 (with different intervals) for anyone older than 65 years [5].

Besides pneumococcal vaccinations, an annual influenza vaccination, as well as, currently, vaccination against SARS-CoV-2, are also very important in preventing morbidity and mortality in patients with lung malignancy [1,6].

The aim of this study was to analyze whether vaccinations in patients with lung cancer are in line with STIKO recommendations. Pneumococcal vaccinations were analyzed in detail.

## 2. Methods

### 2.1. Study Design

The current report is a prospective, single-center carried out study at the University Hospital Regensburg, Germany.

Adherence to vaccination recommendations in lung cancer patients was analyzed in detail by evaluating vaccination certificates, patient medical reports, and a questionnaire [4]. All patients with a scheduled admission to a thoracic-oncological ward or to an oncological outpatient clinic between 1 December 2020 and 29 April 2021 were asked to show their vaccination certificates for review. Patients who possessed no vaccination certificate at all were rated as not vaccinated, patients that failed to present their vaccination certificates were excluded from the study, and in those patients who provided only parts of their existing vaccination certificates, only patients with complete vaccinations were considered for the analysis.

The implementation of STIKO recommendations—which include standard vaccinations that are recommended for all people, indication vaccinations that are recommended for all patients at increased risk of disease, and professional/occupational vaccinations for people with increased risk due to their profession—was analyzed. The study was approved by the Ethics Committee of the University of Regensburg, Germany (reference number 19-1467-2-101).

### 2.2. Patients

Eligibility criteria were applied as follows: histologically proven non-small cell lung cancer (NCSLC) or small cell lung cancer (SCLC), 18 years of age or older, written informed consent, no cognitive impairment, and ability to understand and complete Appendix A.

### 2.3. Statistics

Statistics of continuous variables are presented as mean ± standard deviation. Chi square test and Fisher’s exact test were used for categorical variables. All significance tests were two-tailed. A *p*-value < 0.05 was considered as the threshold for statistical significance. Analyses were performed using Microsoft Excel (version 2016, Microsoft, Redmond, WA, USA) and IBM SPSS (version 24.0, IBM, Armonk, NY, USA).

## 3. Results

### 3.1. Baseline Characteristics

A total of 136 patients (48.5% female) were enrolled (Table 1). Median age was 67.5 years. Non-small cell lung cancer was the predominant histological type (SCLC *n* = 23, 16.9% vs. NSCLC *n* = 113, 83.1%). The majority of patients had metastatic disease (NSCLC stage IV *n* = 90, 66.1%); 98.5% of the patients presented in a good or slightly reduced performance status (ECOG 0-2). The main therapeutic approach was palliative (*n* = 90, 66.1%). Thirty-four (25.0%) patients had pulmonary comorbidities (e.g., COPD, asthma bronchiale, interstitial lung disease). The mean time from diagnosis to study recruitment was 17.8 months (SD 24.1 months).

### 3.2. Vaccination Status

In total, 110 patients (80.9%) brought all, and 9 patients brought incomplete vaccination certificates; 17 patients (12.5%) reported not having a vaccination certificate at all. Following the diagnosis of lung cancer, the vaccination status of 44 patients (32.8%) was reviewed by the general practitioner, and the vaccination status of 4 patients (2.9%) was assessed by a chest physician.

A recommended pneumococcal vaccination (in line with STIKO guidelines, considering the time after initial cancer diagnosis and prior vaccinations) was performed in 9.4% (12/127, Table 1) of the patients. Most patients (79/127, 62.2%) were not vaccinated with any pneumococcal vaccine, and some (36/127, 28.3%) received a pneumococcal vaccination not in line with STIKO recommendations (Table 2). The likelihood of correct vaccination increased significantly (*p* = 0.001) if the vaccination status was reviewed by a medical doctor following the diagnosis of lung cancer. A higher age correlated with a higher adherence to PPSV23 but not to PCV13.

A complete vaccination was performed for tetanus in 50.4% (66/131), diphtheria in 34.4% (44/128), poliomyelitis in 25.8% (33/128), tick-borne encephalitis in 40.7% (24/59), hepatitis A in 45.5% (7/11), hepatitis B in 38.5% (5/13), shingles in 3.0% (3/101), measles in 50.0% (3/6), pertussis in 47.7% (62/130), influenza in 54.4% (74/136), and meningococcal meningitis in 0% (0/2) of the patients (Table A1, Appendix A).

### 3.3. Patients’ Self-Reports

We found that 83 patients (61%) were convinced they had received all recommended vaccinations. In addition, 104 participants (76.4%) were willing to get vaccinated according to STIKO recommendations, and even more patients (113; 83.1%) reported they would like to be vaccinated against SARS-CoV2. Side effects after previous vaccinations were reported by 34 patients (25%), although the reported side effects were mild.

## 4. Discussion

To our knowledge, this is the first study analyzing the vaccination status of lung cancer patients in Southern Germany.

The estimated pneumococcal vaccination rate in our cohort was low (9.4% (12/127)) compared with another that in German study on the vaccination status of patients with pulmonary diseases (29.5%), which was performed before the COVID-19 pandemic [7]. Thus, one possible explanation could be a negative effect of the COVID-19 pandemic, causing the temporary lack of pneumococcal vaccines, according to the Paul Ehrlich Institute, since March 2020. Another reason could be the nescience of physicians. This conclusion is supported by the fact that only 32.8% of the patients had their vaccination status verified following lung cancer diagnosis, which is by far lower than the annual influenza vaccination rate. If in fact, insufficient attention of the treating physicians is related to the inadequate pneumococcal vaccination rate in lung cancer patients, greater effort should be put into an appropriate doctors’ information campaign.

There is also an ongoing debate on the correct strategy of pneumococcal vaccination in patients without neoplasia, which might additionally contribute to uncertainty and a lower vaccination rate [8]. Furthermore, the fear of interference with antineoplastic treatments may impact the adherence to vaccination guidelines.

Overall, 61% of the patients (*n* = 83) believed that their vaccination status was complete, and 76.4% of the study participants (*n* = 104) were willing to receive all vaccinations as recommended. Computer-aided reminder functions might result in a higher adherence to vaccination guidelines. Additionally, a more targeted approach might prove beneficial if oncologists were to supervise and control the vaccination status of their patients. Financial incentives may also be of further help in improving vaccination compliance [9].

A recently published article by our study group demonstrated low adherence to pneumococcal and influenza vaccination in patients with chronic pulmonary disease as well [10]. Insufficient adherence to the recommended vaccinations has also been reported in other studies. For example, in an Italian survey among patients on dialysis, only 57.5% of the participants received the seasonal influenza vaccination [11]. In another German study on the adherence to STIKO recommendations on patients with pulmonary diseases, the influenza vaccination rate was even lower (21%) [7]. In our cohort, the vaccination rate for influenza was also low (54.4%), despite the fact that influenza increases hospitalization and mortality rates in patients with solid tumors [12]. Additionally, many patients with cancer are older and thus even have an indication for influenza vaccination due to their age [1].

In summary, adherence to pneumococcal vaccination, as well as to all other recommended vaccinations, is very low in this study population.

## 5. Limitations

The study presents certain limitations. It was a single-center study, and the cohort comprised only a limited number of patients. Data on vaccination coverage were collected exclusively by assessing the vaccination certificate entries; non-documented vaccinations may thus have been missed. Furthermore, patients who did not show the vaccination certificate (but possessed a vaccination certificate) were excluded. This might have led to an overestimation of the real adherence rate to vaccinations.

## Figures and Tables

**Table 1:** Baseline characteristics (*n* = 136).

Age (Range)	67.5	(37–88)
Gender		
female	66	48.5%
male	70	51.5%
ECOG status		
ECOG 0	88	64.7%
ECOG 1	34	25.0%
ECOG 2	12	8.8%
ECOG 3	2	1.5%
ECOG 4	0	0.0%
Disease		
SCLC	23	16.9%
NSCLC	113	83.1%
Therapeutic approach		
curative	46	66.1%
palliative	90	33.9%
Pneumococcal vaccination		
any vaccination against pneumococcus	51/130	39.2%
any vaccination with PCV13	16/127	12.6%
any vaccination with PPSV23	43/130	33.1%
vaccinated in line with STIKO recommendations	12/127	9.4%
vaccinated not in line with STIKO recommendations	36/127	28.3%
no vaccination at all	79/127	62.2%

ECOG: Eastern Co-operative Oncology Group. SCLC: small cell lung cancer. NSCLC: non-small cell lung cancer. STIKO: German Standing Committee on Vaccination.

**Table 2:** Deviation from STIKO recommendations in patients who received a pneumococcal vaccination.

PCV13	PPSV23	Number
Not Vaccinated in Line with STIKO Recommendations		
yes	yes >72 months prior to presentation	2.4% (3/127)
yes, vaccination prior to diagnosis and >12 months between vaccination and presentation	no	1.6% (2/127)
no	yes, prior to diagnosis and >72 months prior to presentation	3.9% (5/127)
no	yes, prior to diagnosis and >12 months prior to presentation	8.7% (11/127)
yes, vaccination after diagnosis and >12 months between vaccination and presentation	no	0.8% (1/127)
no	yes, after diagnosis	11.0% (14/127)
		**28.3%** **(36/127)**

## Data Availability

Authors will respond to data sharing requests under the premise that an adequate research question is formulated. Original anonymized data will be made available up to one year after the publication of the paper.

## Appendix A

**Table A1:** Results from the vaccination certificates All vaccinations certificates were available from 110 patients; from 9 patients, at least some information was included, while 17 patients stated that they did not have a vaccination certificate, so these patients were considered as not vaccinated (according to STIKO recommendations). Patients who provided only part of their existing vaccination certificates were considered for the analysis in the case of complete vaccination. Since indications for immunizations vary for different vaccines, every vaccine is listed separately. Shading of cells was used to show that no STIKO recommendation exists.

Vaccination	Standard Vaccination (S)—For Universal Application	Indication Vaccination (I)—For Increased Risk (Rather than Professional)	Occupational Vaccination (O)—For Increased Occupational Risk	Any Indication	Vaccination Without Current Risk Profile/Vaccination in Infancy/Travel Vaccination
Diphtheria	34.3% (44/128)			34.4% (44/128)	
Hemophilus influenza type b		0 patients		0 patients	0% (0/127)
Hepatitis A		37.5% (3/8)	66.7% (2/3)	45.5% (5/11)	15.4% (18/117)
Hepatitis B		25.0% (2/8)	60.0% (3/5)	38.5% (5/13)	15.7% (18/115)
Human papilloma virus				0 patients	0.0% (0/127)
Influenza	58.5% (55/94)	54.4% (74/136)	45.0% (9/20)	54.4% (74/136)	0 patients
Measles	50.0% (3/6)	0 patients	0 patients	50.0% (3/6)	4.1% (5/123)
Meningococcal		0.0% (0/2)	0 patients	0.0% (0/2)	ACWY: 0.8% (1/125) MenB: 0.0% (0/125)
Mumps			0 patients	0 patients	1.6% (2/128)
Pertussis	47.7% (62/130)	60.0% (3/5)	57.1% (8/14)	47.7% (62/130)	
Pneumococcal	41.8% (38/91)	9.4% (12/127)	0.0% (0/3)	9.4% (12/127)	0 patients
Poliomyelitis	25.8% (33/128)	0 patients	0.0% (0/2)	25.8% (33/128)	
Rabies			0 patients	0 patients	0.8% (1/127)
Rubella		0 patients	0 patients	0 patients	3.9% (5/129)
Shingles	3.4% (3/88)	3.9% (2/51)		3.0% (3/101)	0.0% (0/26)
Tetanus	50.4% (66/131)			50.4% (66/131)	
Tick-borne encephalitis		40.7% (24/59)	0 patients	40.7% (24/59)	19.4% (14/72)
Varicella		0 patients	0 patients	0 patients	0.8% (1/128)
Yellow fever			0 patients	0 patients	n.a.

## References

[1] Rieger C.T., Liss B., Mellinghoff S., Buchheidt D., Cornely O.A., Egerer G., Heinz W.J., Hentrich M., Maschmeyer G., Mayer K. (2018). Anti-infective vaccination strategies in patients with hematologic malignancies or solid tumors-Guideline of the Infectious Diseases Working Party (AGIHO) of the German Society for Hematology and Medical Oncology (DGHO). Ann. Oncol..

[2] Siegel R., Ma J., Zou Z., Jemal A. (2014). Cancer statistics, 2014. CA Cancer J. Clin..

[3] GBD 2016 Lower Respiratory Infections Collaborators (2018). Estimates of the global, regional, and national morbidity, mortality, and aetiologies of lower respiratory infections in 195 countries, 1990–2016: A systematic analysis for the Global Burden of Disease Study 2016. Lancet.

[4] Kling K., Koch J., Vygen-Bonnet S., Wichmann O. (2021). Empfehlungen der Ständigen Impfkommission (STIKO) am Robert Koch-Institut 2020/2021.

[5] Mohr A., Kloos M., Schulz C., Pfeifer M., Salzberger B., Bauernfeind S., Hitzenbichler F., Plentz A., Koch M. (2020). Low adherence to pneumococcal vaccination in lung cancer patients in a tertiary care university hospital in Southern Germany. Epi. Bull..

[6] CDC CfDCaP (2019). Pneumococcal Vaccination. https://www.cdc.gov/vaccines/vpd/pneumo/index.html.

[7] Luo J., Rizvi H., Preeshagul I.R., Egger J.V., Hoyos D., Bandlamudi C., McCarthy C.G., Falcon C.J., Schoenfeld A.J., Arbour K.C. (2020). COVID-19 in patients with lung cancer. Ann. Oncol..

[8] Mohr A., Plentz A., Sieroslawski A., Pezenburg F., Koch M., Bauernfeind S., Pfeifer M., Salzberger B., Hitzenbichler F. (2021). Adherence to STIKO recommendations in patients with pulmonary disease in southeast Germany. Infection.

[9] Jackson L.A., Gurtman A., van Cleeff M., Jansen K.U., Jayawardene D., Devlin C., Scott D.A., Emini E.A., Gruber W.C., Schmoele-Thoma B. (2013). Immunogenicity and safety of a 13-valent pneumococcal conjugate vaccine compared to a 23-valent pneumococcal polysaccharide vaccine in pneumococcal vaccine-naive adults. Vaccine.

[10] Sanftenberg L., Brombacher F., Schelling J., Klug S.J., Gensichen J. (2019). Increasing Influenza Vaccination Rates in People with Chronic Illness. Dtsch. Ärztebl. Int..

[11] Mohr A., Plentz A., Sieroslawski A., Pezenburg F., Pfeifer M., Salzberger B., Hitzenbichler F. (2020). Use of Pneumococcal and influenza vaccine in patients with COPD, asthma bronchiale and interstitial lung diseases in Southeast Germany. Respir. Med..

[12] Battistella C., Quattrin R., Celotto D., d’Angelo M., Fabbro E., Brusaferro S., Agodi A., Astengo M., Baldo V., Baldovin T. (2019). Factors predicting influenza vaccination adherence among patients in dialysis: An Italian survey. Hum. Vaccines Immunother..

